# Chronic intermittent tachycardia as a consequence of vagus nerve injury after anterior cervical discectomy and fusion: case report of a previously unreported complication

**DOI:** 10.1016/j.xnsj.2023.100291

**Published:** 2023-11-04

**Authors:** Erik Werheim, Zachary Sokol, Neha Mody, Justice Owusu-Agyei

**Affiliations:** a Department of Neurosurgery, St Luke's University Health Network, 801 Ostrum St. Bethlehem, PA 18015, United States; b Department of Neurosurgery, Lewis Katz School of Medicine at Temple University, 3401 N Broad St, Philadelphia, PA 19140, United States

**Keywords:** Anterior cervical discectomy and fusion, Vagus nerve, Complications, Tachycardia, ACDF, Cervical

## Abstract

**Background:**

The surgical approach of an anterior cervical discectomy and fusion (ACDF) navigates many important neurologic and vascular structures in the neck. More frequently reported complications are dysphagia, postoperative hematoma, cerebrospinal fluid leaks, and dysphonia.

**Case description:**

This case report details an ACDF in a 49-year-old female with intractable neck pain and radicular symptoms. Following the procedure, she developed intermittent tachycardia at rest, which worsened with exertion.

**Outcome:**

The cardiac workup was negative. A neck ultrasound demonstrated hypoechoic thickening of the vagus nerve, providing the diagnosis of vagus nerve injury. The patient's tachycardia has been managed with beta-blockers.

**Conclusions:**

Although previously unreported, vagus nerve injury following ACDF is possible, causing sympathetic disruption, which can be managed with beta blockers.

## Introduction

Anterior Cervical Discectomy and Fusion (ACDF) is one of the most common neurosurgical procedures with rates increasing significantly throughout the years. From 1990 to 1999 over 500,000 ACDF procedures were performed and over one million ACDF procedures were performed from 2006 to 2013 [1,2]. During ACDF procedures, a prudent approach to the identification of important anatomical landmarks and their corresponding structures is paramount to a successful outcome [3].

Although complications are rare, some of the more frequently reported are dysphagia, postoperative wound hematoma, recurrent laryngeal nerve palsy (RLN), adjacent segment disc disease, and intra-op cerebrospinal fluid leaks. Complications such as esophageal perforation, sympathetic denervation leading to Horner's Syndrome, and vascular injury (carotid, vertebral, jugular) are reported less frequently [4], [5], [6]. In this case study, we report a complication of chronic intermittent tachycardia secondary to vagal nerve injury in a 49-year-old female who underwent ACDF.

## Case report

### Patient info

A 49-year-old female with a past medical history of fibromyalgia and rheumatoid arthritis presented with acute worsening of chronic neck pain. She had exhausted conservative treatment options. The pain, described as burning in nature, radiated from her paraspinal muscles up the back of her head, down her shoulders and arms bilaterally, and occasionally into her fingertips. She noted decreased strength and dexterity. Her symptoms were affecting her sleep and daily routines.

Neurologic and musculoskeletal examination noted a positive Spurling's sign on the left, paravertebral spasm, and restriction in cervical range of motion. She had intact strength in the upper and lower extremities and sensation to light touch and pinprick diffusely. Hoffman's sign was negative, and no ankle clonus was present.

Computed tomography (CT) detected cervical spondylosis with partially calcified disc herniations from C4 to C7 resulting in central canal stenosis and left C5–C6 foraminal stenosis. Minimal calcification of the posterior longitudinal ligament was evident.

**Fig. 1:**
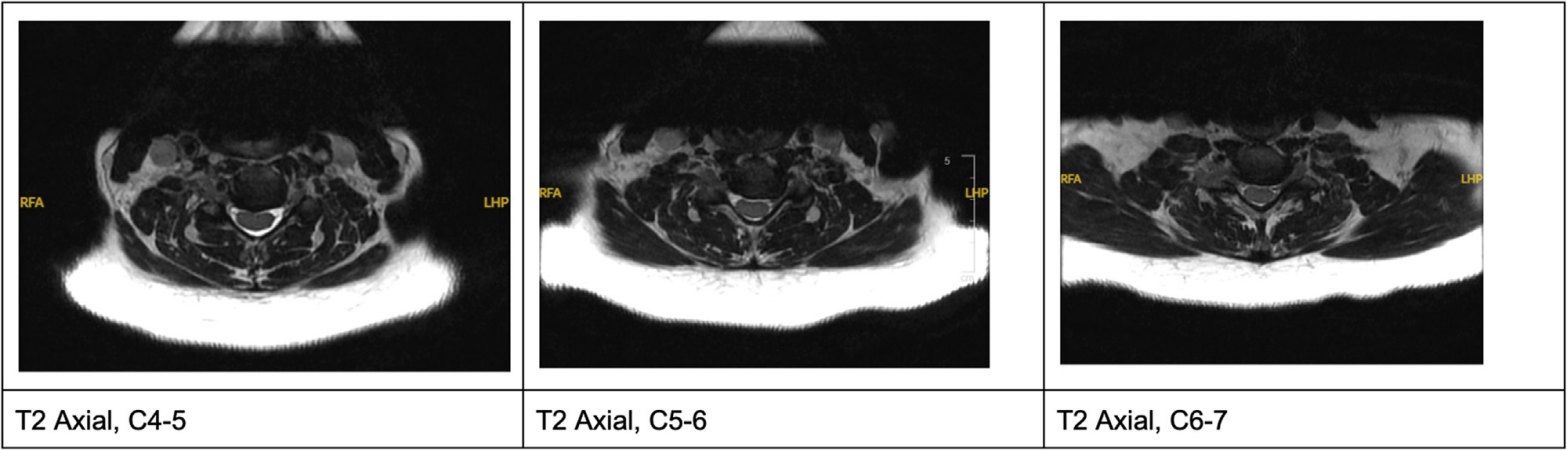
Preop T2 Axials demonstrating disc herniations with central canal stenosis throughout, with severe left C5-6 foraminal stenosis.

**Fig. 2:**
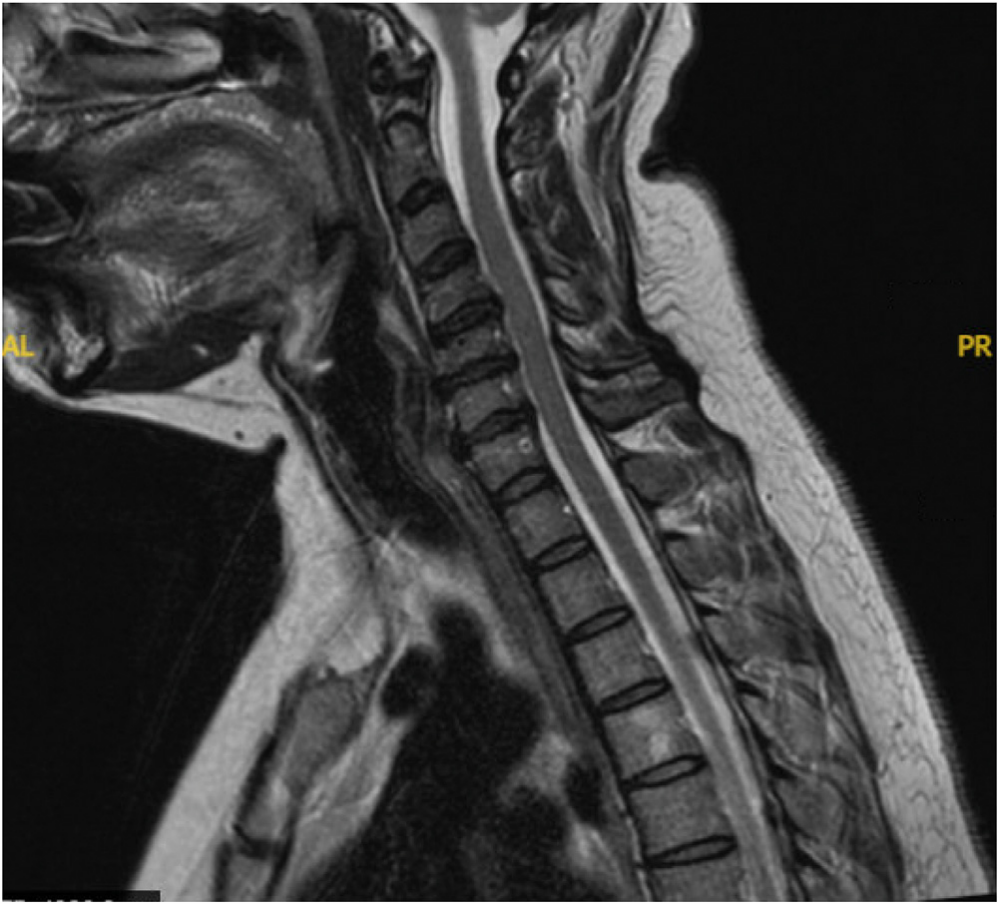
Preop T2 Sagittal demonstrating disc hernation with central canal stenosis at C4–5, C5–6, C6–7.

Magnetic resonance imaging (MRI) displayed diffuse degeneration of C4–C7 with disc herniations throughout and an osteophyte complex at C6–C7. Moderate central canal narrowing was evident throughout the mentioned levels as well as severe left foraminal narrowing of C5–C6 (Fig. 1, Fig. 2).

The patient underwent an ACDF of levels C4–C7 through a small incision in the skin crease on the right side. The platysma was incised and the sternocleidomastoid muscle was identified. The medial avascular plane was bluntly dissected. Cloward retractors were used to retract the esophagus and trachea medially and the carotid artery-jugular complex was retracted laterally; revealing the prevertebral fascia and the longus coli muscles. A Thompson-Farley retractor was used to optimize visualization and care was taken to avoid injury to adjacent anatomical structures. The vagus nerve was never encountered. After level verification, discectomy was performed at C4–5, C5–6, and C6–7. The discectomy and the rest of the procedure were performed with the microscope. The posterior longitudinal ligament was identified and cut transversely using Kerrison rongeurs. Interbody cages were then placed followed by anterior plating and screws. A drain was placed, and the wound was closed in standard fashion. Neuromonitoring was utilized during the case, with both somatosensory and motor-evoked potentials. No significant deviations from the baseline were encountered during the course of the operation. From incision to closure, the case lasted approximately 175 minutes.

The patient's hospital course after the procedure was uncomplicated. Her original neurological symptoms improved. Her main complaints were expected postoperative pain, muscle spasm, and swallowing discomfort, which all improved significantly a few days after. However, she experienced periodic episodes of sustained sinus tachycardia ranging from heart rates in the 100 to 110 seconds while resting and with spikes up to 150 seconds with exertion prompting a cardiology consult. Fluids were given to correct any hypovolemia, EKG and troponins were unremarkable aside from sinus tachycardia, TSH was within normal limits, and computed tomography and angiography (CTA) ruled out pulmonary embolism. There was no evidence of fever or leukocytosis suggesting an infection. She did not have any evidence of sympathetic injury, nor did she have any other signs of vagus nerve injury. She was eventually discharged home on postop day 4. A review of the patient's pre-operative MRI did not reveal any abnormalities of the carotid sheath or vagus nerve.

**Fig. 3:**
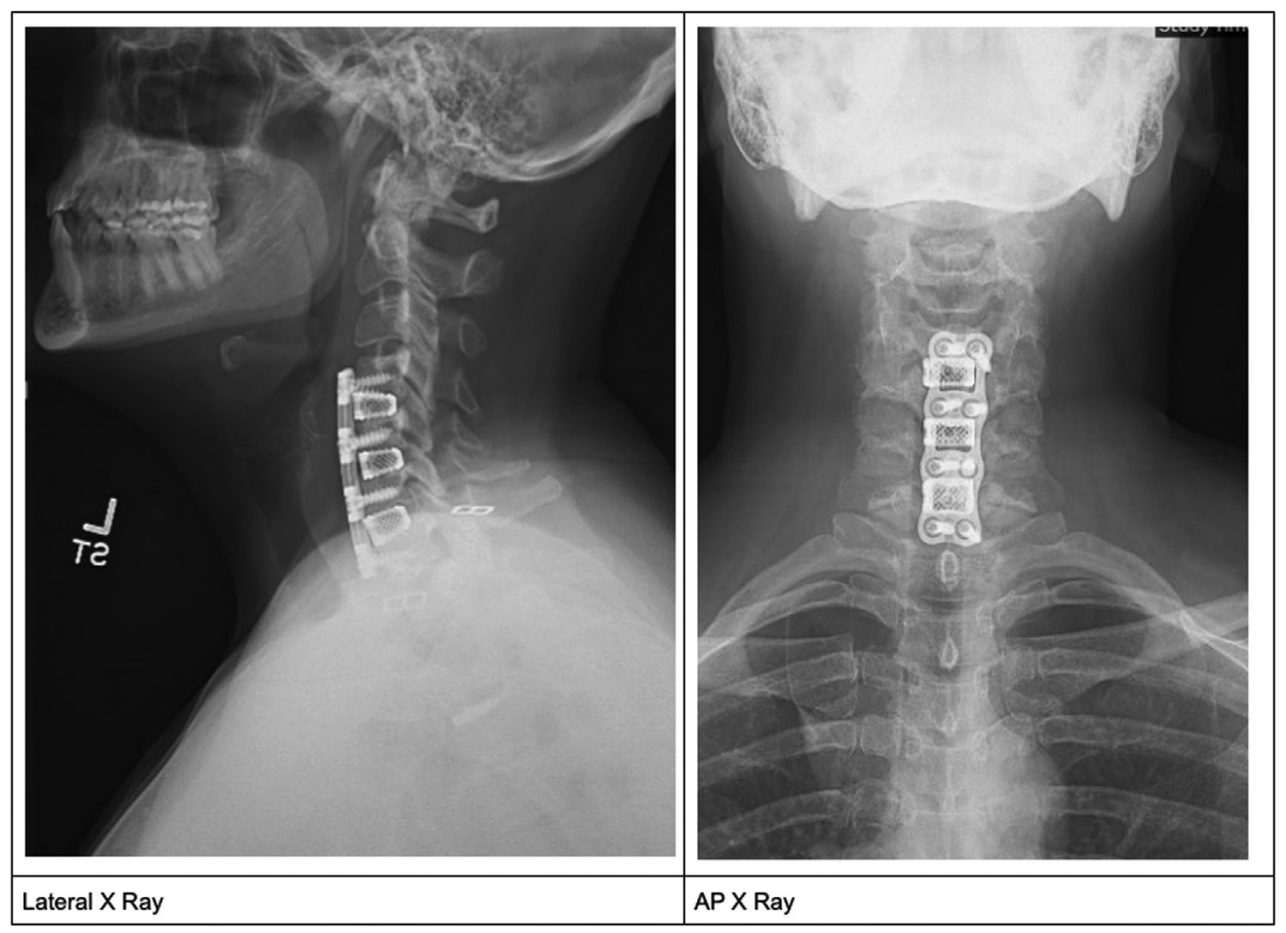
Postop lateral and AP X-ray demonstrating intact hardware at C4–7

The patient continued to follow up with cardiology and neurosurgery. Follow-up imaging did not demonstrate any hardware failure (Fig. 3). In the months following the operation, she continued to experience heart rates in the range of 100 to 130 along with some episodes of lightheadedness. She was started on metoprolol XL 25 mg twice a day, which helped lower her resting heart rates into the *60s* to *80s*, however, sinus tachycardia would still occur with light exertion and other events such as eating. The patient underwent a vagus nerve ultrasound demonstrated a thickened and hypoechoic vagus nerve one inch inferior to the skin incision. The nerve is contiguous with no hyperemia. The findings suggest neuropathy perhaps due to local scar tissue formation.

## Discussion

Understanding the anatomy of the cervical region helps improve surgical outcomes and lower complication rates in ACDF procedures. The common carotid artery bifurcation typically occurs around C4, the facial vein draining into the internal jugular vein (IJV) is usually around C3–4 but can be variable, and the vertebral arteries enter the transverse foramina at C6 [3]. The cervical sympathetic chain lying over the longus coli and longus capitus muscles is important to note during retraction and exposure of the prevertebral fascia. The recurrent laryngeal nerve traveling with the omohyoid muscle and the carotid sheath containing the internal jugular vein, vagus nerve, and internal carotid are all vital structures to consider during an ACDF approach [3].

Some of the complications of ACDF procedures are due to excessive or prolonged traction of these neurologic and anatomic structures. Dysphagia is typically a result of esophageal retraction. From a neurologic standpoint, hoarseness, or dysphonia from RLN palsy can be due to excessive traction of either the omohyoid muscle or tracheoesophageal groove, depending on the side of approach. There have been rare instances of Horner's Syndrome as well from excessive traction on the longus muscles [4], [5], [6], [7]. An incidence of bilateral phrenic nerve palsy was reported after ACDF from C4 nerve root stretching [8]. Vascular complication is less commonly reported, aside from postoperative hematoma. However, a report of IJV thrombosis has been published following an ACDF thought to be the result of retraction and manipulation of the IJV [9]. There has never previously been a report of vagus nerve injury resulting in sustained tachycardia following an ACDF.

The connection between the vagus nerve and its parasympathetic innervation of the cardiovascular system has been well-established and documented [10]. One function of parasympathetic stimulation is the reduction of a person's heart rate. Since volume derangements were corrected, and non-neurologic causes were ruled out, we postulate that the tachycardia following the ACDF procedure was due to vagal injury and denervation. The mechanism of the denervation is most likely the retraction of the sternocleidomastoid and the carotid sheath, housing the vagus nerve. We believe this is the most logical mechanism, as retraction and manipulation are commonly reported as the culprits of other complications in literature [4], [5], [6], [7], [8], [9]. This report furthers the emphasis needed on monitoring the amount of traction put on important anatomical structures during an operation. Although other neurologic injuries are more frequently reported, the vagus nerve should be considered in the differential of autonomic abnormalities following an ACDF procedure.

## Conclusion

ACDF is one of the most frequently performed neurosurgical procedures. During exposure to the anterior aspect of the vertebral bodies, careful consideration for important anatomical landmarks is vital to the operation's success and the minimization of complications. Symptoms such as dysphonia, hoarseness, respiratory insufficiency, or a Horner's presentation following ACDF should bring on suspicion of possible nervous injury.

While some of these symptoms may be transient, others can persist and warrant further medical management. Important identification and intervention can aid in the management and recovery of such complications. This case report depicts a patient with sustained tachycardia following an ACDF from a vagal nerve injury.

Patient consent statement

The patient provided informed consent for publication of this case report.

## Declaration of Competing Interest

The authors declare that they have no known competing financial interests or personal relationships that could have appeared to influence the work reported in this paper.
